# Post-Hemorrhagic Hydrocephalus with Secondary Cerebrospinal Fluid Pathway Obstruction in an Extremely Premature Infant: A Case Report

**DOI:** 10.3390/children13070860

**Published:** 2026-06-28

**Authors:** Ahmad Kharoufeh, Mohammed Dalbah, Haidy Alzaghal, Malak Abedi, Subhranshu Sekhar Kar, Mohamed Anas Patni, Rajani Dube, Tanya Densil, Hussein Eleimy

**Affiliations:** 1 RAK College of Medical Sciences, Ras Al Khaimah Medical and Health Sciences University, Ras Al Khaimah P.O. Box 11172, United Arab Emirates; mohammed.20901105@rakmhsu.ac.ae (M.D.); haidy.24901036@rakmhsu.ac.ae (H.A.); malaek.21901100@rakmhsu.ac.ae (M.A.); tanya.21901069@rakmhsu.ac.ae (T.D.); 2 Department of Pediatrics and Neonatology, RAK College of Medical Sciences, Ras Al Khaimah Medical and Health Sciences University, Ras Al Khaimah P.O. Box 11172, United Arab Emirates; 3 Department of Community Medicine, RAK College of Medical Sciences, Ras Al Khaimah Medical and Health Sciences University, Ras Al Khaimah P.O. Box 11172, United Arab Emirates; mohamedanas@rakmhsu.ac.ae; 4 Department of Obstetrics and Gynecology, RAK College of Medical Sciences, Ras Al Khaimah Medical and Health Sciences University, Ras Al Khaimah P.O. Box 11172, United Arab Emirates; rajani.dube@rakmhsu.ac.ae; 5 Department of Pediatrics, Fujairah Hospital, Fujairah P.O. Box 11172, United Arab Emirates; hussien.eleimy@ehs.gov.ae

**Keywords:** post-hemorrhagic hydrocephalus, extreme prematurity, intraventricular hemorrhage, neonatal neuroimaging, ventriculoperitoneal shunt, neurodevelopmental outcome

## Abstract

**Highlights:**

**What are the main findings?**

- Initially documented Grade II intraventricular hemorrhage progressed to severe shunt-dependent post-hemorrhagic ventricular dilatation.
- Serial neuroimaging demonstrated imaging features suggestive—but not diagnostic—of a non-communicating hydrocephalus pattern.

**What is the implication of the main finding?**

- Even apparently low-grade intraventricular hemorrhage requires careful serial neuroimaging because severe PHVD may still develop.
- Early recognition and timely neurosurgical intervention are essential to optimize clinical outcomes.

**Abstract:**

Background/Objectives: Post-hemorrhagic hydrocephalus (PHH) is a major complication of extreme prematurity associated with significant neurodevelopmental morbidity. Although post-hemorrhagic ventricular dilatation (PHVD) is a recognized consequence of intraventricular hemorrhage (IVH), progression from an apparently low-grade IVH to severe shunt-dependent disease with imaging features suggestive—but not diagnostic—of a non-communicating hydrocephalus pattern is uncommon and presents important diagnostic and management challenges. We report such a case. Case Presentation: A male infant born at 26 weeks’ gestation developed an initially documented Grade II intraventricular hemorrhage that subsequently evolved into progressive post-hemorrhagic ventricular dilatation. Serial cranial ultrasonography demonstrated progressive ventriculomegaly, later confirmed by computed tomography showing marked dilatation of the lateral and third ventricles with severe cortical mantle thinning and a relatively preserved fourth ventricle, raising suspicion of a non-communicating hydrocephalus pattern. Clinical deterioration with progressive macrocephaly and neurological manifestations necessitated temporizing ventricular cerebrospinal fluid drainage, followed by ventriculoperitoneal shunt placement after stabilization and management of secondary complications. Management throughout the clinical course relied on serial neuroimaging, multidisciplinary decision-making, and individualized neurosurgical intervention. Conclusions: This case illustrates that an apparently low-grade neonatal intraventricular hemorrhage may evolve into severe shunt-dependent PHVD and emphasizes the importance of serial neuroimaging surveillance, objective assessment of ventricular progression, and cautious interpretation of imaging findings suggestive—but not diagnostic—of a non-communicating hydrocephalus pattern. It further highlights the diagnostic and therapeutic challenges encountered when atypical radiological evolution complicates the management of PHVD in extremely premature infants.

## 1. Background

Hydrocephalus is one of the most serious neurological complications affecting extremely premature infants and most commonly develops as a sequela of intraventricular hemorrhage (IVH) [1,2]. The germinal matrix vasculature of preterm neonates is particularly fragile and susceptible to fluctuations in cerebral blood flow and oxygenation, making IVH especially common during the first days of life. When hemorrhage extends into the ventricular system, blood products and inflammatory debris may disrupt normal cerebrospinal fluid (CSF) circulation and absorption, resulting in post-hemorrhagic ventricular dilatation (PHVD) and, in some infants, post-hemorrhagic hydrocephalus (PHH) [3]. The underlying mechanism is multifactorial and may involve impaired CSF absorption as well as obstruction of CSF pathways by hemorrhagic debris. Progressive ventricular enlargement increases intracranial pressure, compresses the periventricular white matter, disrupts cortical development, and may lead to long-term neurological sequelae including cerebral palsy, developmental delay, and visual impairment. Neurological outcome is influenced by both the severity of ventricular enlargement and the timing of intervention [4,5]. Serial cranial ultrasonography remains the preferred bedside modality for monitoring ventricular size in preterm infants, while computed tomography (CT) and magnetic resonance imaging (MRI) may provide additional anatomical information when ventricular enlargement or secondary CSF pathway obstruction is suspected. Management ranges from temporizing CSF diversion procedures to definitive ventriculoperitoneal (VP) shunt placement once the infant is clinically suitable and infection has been excluded [6]. Although PHVD is a recognized complication of IVH, progression from an apparently low-grade hemorrhage to severe shunt-dependent disease with imaging features suggestive but not diagnostic of a non-communicating hydrocephalus pattern is uncommon and may create important diagnostic and management challenges. We therefore present this case to illustrate the atypical clinical and radiological evolution of PHVD and the multidisciplinary decision-making required throughout its management.

## 2. Case Presentation

Cranial ultrasonography performed during the neonatal admission demonstrated an initially documented Grade II intraventricular hemorrhage. Serial follow-up ultrasonographic examinations subsequently showed progressive post-hemorrhagic ventricular dilatation, with gradual enlargement of the lateral and third ventricles, indicating ongoing ventricular expansion despite continued clinical surveillance. Brain MRI performed on 21 May 2025 demonstrated bilateral parieto-temporal subcortical/periventricular cystic encephalomalacia, a right frontoparietal intracerebral subacute hematoma, moderately dilated supratentorial ventricles, and intraventricular hemosiderin deposition consistent with sequelae of previous intraventricular hemorrhage. No dedicated MRI cerebrospinal fluid flow study was performed.

**Figure 1:**
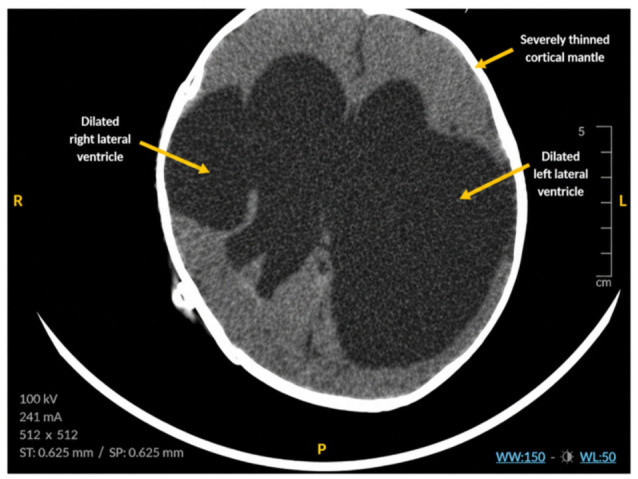
Axial non-contrast CT demonstrating marked dilatation of the lateral ventricles with severe cortical mantle thinning, consistent with advanced ventriculomegaly. R = right; L = left; P = posterior.

As ventricular enlargement and head circumference continued to increase, computed tomography (CT) was obtained on 29 September 2025 (approximately 50 weeks postmenstrual age) as part of urgent neurosurgical evaluation. CT confirmed severe ventriculomegaly with marked dilatation of the lateral and third ventricles accompanied by pronounced cortical mantle thinning (Figure 1).

**Figure 2:**
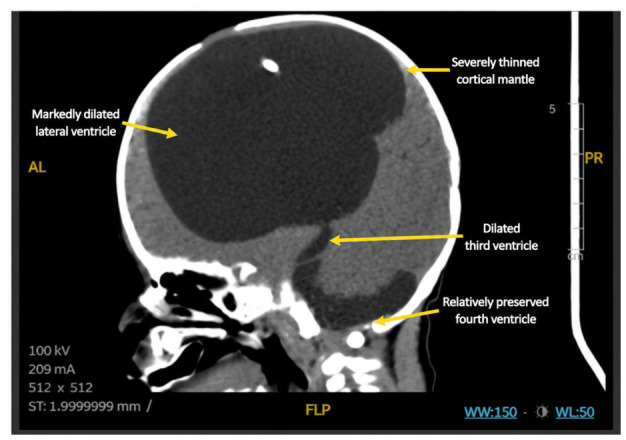
Sagittal CT reconstruction demonstrating marked enlargement of the lateral and third ventricles with relative preservation of the fourth ventricle (arrows), producing an imaging pattern suggestive—but not diagnostic—of a non-communicating hydrocephalus pattern.

Sagittal CT reconstruction (Figure 2) demonstrated relative preservation of the fourth ventricle despite marked enlargement of the lateral and third ventricles. This radiological pattern raised suspicion of a non-communicating hydrocephalus pattern secondary to intraventricular hemorrhage; however, CT alone was insufficient to establish the exact level or mechanism of cerebrospinal fluid pathway obstruction.

**Figure 3:**
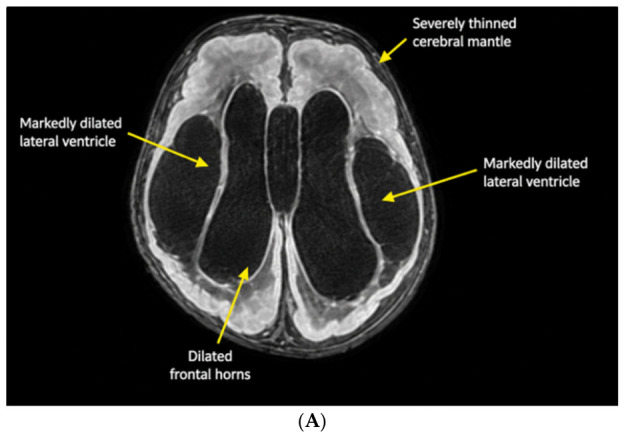

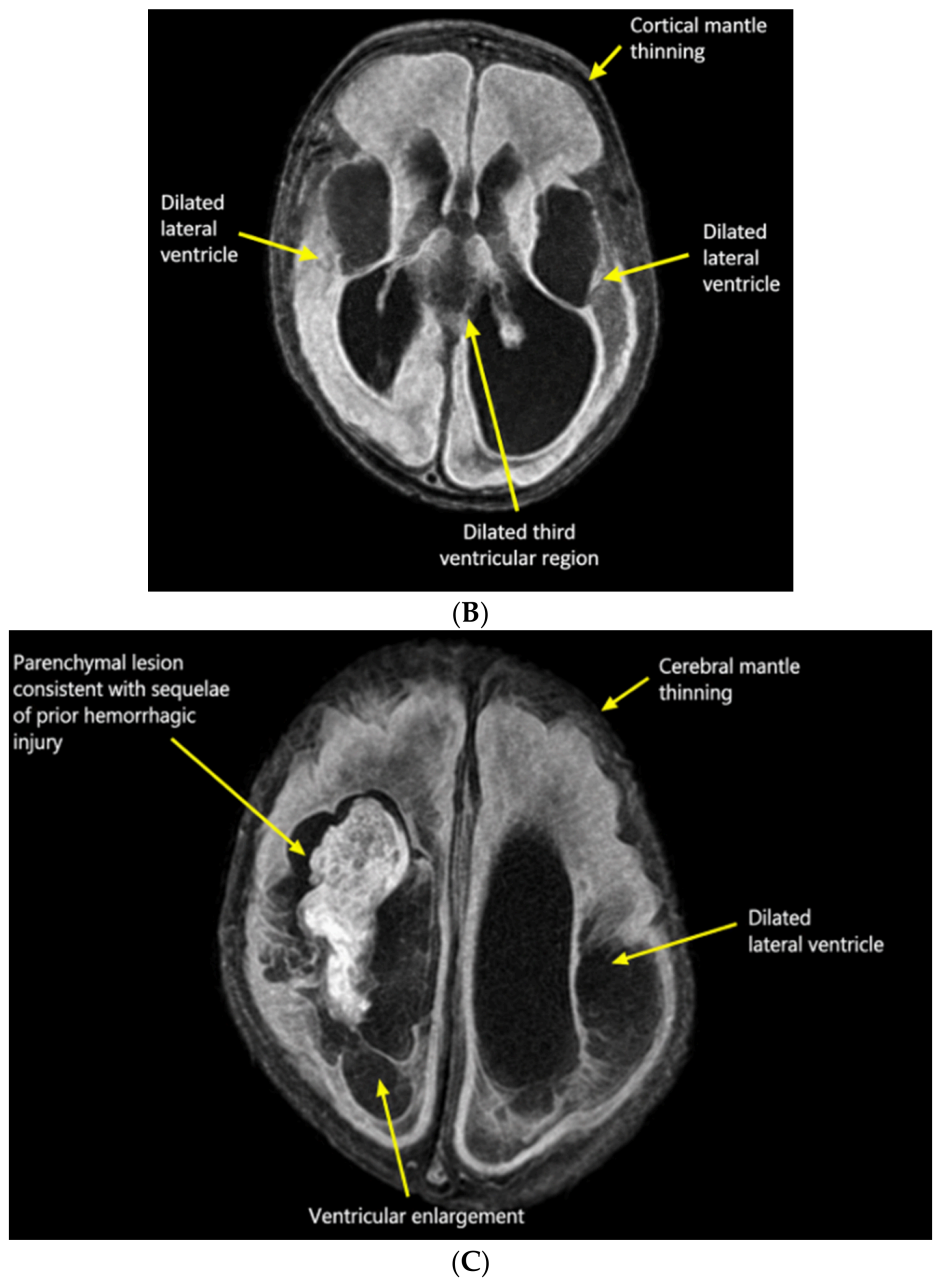
Representative brain MRI images obtained on 21 May 2025. (**A**) Severe ventriculomegaly with marked dilatation of the lateral ventricles and pronounced cerebral mantle thinning. (**B**) Additional axial section confirming extensive supratentorial ventricular enlargement with distortion of adjacent cerebral structures. (**C**) Associated parenchymal abnormalities, including a right frontoparietal hemorrhagic lesion and surrounding changes consistent with sequelae of prior hemorrhagic brain injury in the setting of neonatal intraventricular hemorrhage. The MRI findings demonstrated severe ventricular enlargement, intraventricular hemorrhagic sequelae, and associated parenchymal injury. However, because dedicated cerebrospinal fluid flow sequences were not performed, MRI could not definitively establish the exact site or mechanism of cerebrospinal fluid pathway obstruction. (**A**) Axial brain MRI demonstrating marked bilateral ventriculomegaly with severe dilatation of the lateral ventricles, enlargement of the frontal horns, and pronounced thinning of the surrounding cerebral mantle. (**B**) Additional axial brain MRI section demonstrating marked enlargement of the supratentorial ventricular system, including severe bilateral lateral ventricular dilatation, distortion of adjacent cerebral structures, and associated cerebral mantle thinning consistent with advanced post-hemorrhagic ventricular dilatation. (**C**) Axial MRI demonstrating parenchymal abnormalities adjacent to the ventricular system, including a right frontoparietal hemorrhagic lesion and surrounding changes consistent with sequelae of prior hemorrhagic brain injury in the setting of neonatal intraventricular hemorrhage.

Therefore, these imaging findings should be interpreted as radiological suspicion rather than confirmation of a non-communicating hydrocephalus pattern. Brain MRI performed earlier in the clinical course demonstrated ventriculomegaly and sequelae of prior intraventricular hemorrhage, including intraventricular hemosiderin deposition; however, MRI cerebrospinal fluid flow studies were not performed (Figure 3). Consequently, definitive localization of the site or mechanism of cerebrospinal fluid pathway obstruction could not be established and would have required dedicated CSF flow MRI or endoscopic assessment.

Cranial ultrasonography remained the primary modality for serial monitoring throughout the infant’s clinical course. Serial head circumference measurements demonstrated progressive macrocephaly, increasing from 26 cm on day 3 of life to 40 cm by 29 September 2025 and subsequently reaching 42–43 cm during follow-up, exceeding the 97th percentile for age. This progression was accompanied by irritability, poor feeding, intermittent vomiting, and neurological findings including a full anterior fontanelle, increased muscle tone, and bilateral ankle clonus, raising concern for progressive intracranial hypertension.

Given the combined clinical deterioration and radiological progression, consistent with increasing intracranial pressure, a ventricular tap was performed as a temporizing intervention for both therapeutic decompression and diagnostic evaluation. Approximately 60 mL of clear cerebrospinal fluid was removed during the procedure. At the time of intervention, the infant weighed approximately 4.8–5.0 kg, and the volume removed was determined by the treating neurosurgical team based on the severity of ventriculomegaly, clinical evidence of raised intracranial pressure, and the infant’s body weight. The procedure resulted in transient clinical improvement, with increased alertness and improved feeding.

Despite temporary clinical improvement following ventricular tapping, progressive ventriculomegaly and continued enlargement of head circumference persisted. Consequently, a ventriculoperitoneal shunt was inserted on 6 October 2025 as definitive cerebrospinal fluid diversion because of persistent progressive ventriculomegaly, increasing head circumference, clinical evidence of raised intracranial pressure, and ongoing dependence on cerebrospinal fluid diversion.

The subsequent clinical course was complicated by VP shunt malfunction and a central nervous system infection. The patient was re-admitted on 26 November 2025 with progressive irritability, increasing head circumference, and concern for shunt-related complications. Cerebrospinal fluid analysis demonstrated a glucose concentration of **1.2 mmol/L** and a protein concentration of **2175 mg/L**, and cerebrospinal fluid culture grew *Staphylococcus lugdunensis*. The patient initially received intravenous ceftriaxone from 28 November to 5 December 2025. Following microbiological confirmation and susceptibility testing, antimicrobial therapy was changed to culture-directed intravenous vancomycin (15 mg/kg/dose every 12 h), which was continued until 18 December 2025. Clinical and laboratory improvement was observed during treatment.

During the same hospitalization, VP shunt malfunction was identified, and VP shunt revision was performed on 28 November 2025. Operative findings demonstrated partial obstruction of the ventricular catheter, necessitating revision of the cranial and abdominal components of the shunt. Following shunt revision, completion of antimicrobial therapy, and clinical stabilization, satisfactory cerebrospinal fluid drainage was restored.

The clinical course was further complicated by the patient’s extreme prematurity, progressive post-hemorrhagic ventricular dilatation, and shunt-related complications, requiring multidisciplinary management involving neonatology, neurosurgery, radiology, infectious diseases, and developmental pediatrics. The patient was discharged on 16 December 2025 in stable clinical condition with a functioning revised ventriculoperitoneal shunt and scheduled follow-up with neurosurgery and developmental pediatrics for ongoing assessment of shunt function and neurological development. At the time of manuscript preparation, long-term neurodevelopmental outcome data were not yet available. The patient’s clinical course is summarized in Table 1.

## 3. Discussion

Post-hemorrhagic ventricular dilatation (PHVD) and post-hemorrhagic hydrocephalus (PHH) remain important neurological complications of extreme prematurity, resulting from intraventricular hemorrhage and subsequent disruption of cerebrospinal fluid (CSF) circulation and absorption. In the present case, the clinically important feature was not simply the occurrence of PHVD, but the progression from an initially documented Grade II intraventricular hemorrhage to severe shunt-dependent ventricular dilatation with imaging features suggestive—but not diagnostic—of a non-communicating hydrocephalus pattern.

Severe PHVD is more commonly associated with higher-grade intraventricular hemorrhage. Therefore, progression following an initially low-grade hemorrhage warrants careful interpretation. Several mechanisms may explain this evolution, including organization of intraventricular blood products, inflammatory-mediated fibrosis, impaired CSF absorption, and secondary obstruction of CSF pathways. In addition, the initial hemorrhagic burden may occasionally be underestimated on early ultrasonography, or the hemorrhagic process may evolve after the initial grading. In this patient, serial imaging documented progressive ventriculomegaly despite the initial Grade II classification, emphasizing that continued neuroimaging surveillance remains important even when the initial hemorrhage appears less severe. Previous studies have also highlighted that the timing of intervention and progression of ventricular dilatation may influence subsequent neurodevelopmental outcomes in affected infants [7,8,9].

The imaging findings in this case required cautious interpretation. Brain MRI performed earlier in the clinical course demonstrated bilateral parieto-temporal subcortical and periventricular cystic encephalomalacia, a right frontoparietal intracerebral subacute hematoma, moderately dilated supratentorial ventricles, and intraventricular hemosiderin deposition consistent with prior intraventricular hemorrhage. These MRI findings provided additional evidence of chronic hemorrhagic brain injury and supported the interpretation that the ventricular enlargement evolved in the context of post-hemorrhagic disease rather than isolated congenital hydrocephalus. Subsequent CT imaging demonstrated marked dilatation of the lateral and third ventricles with severe cortical mantle thinning and relative preservation of the fourth ventricle. This radiological pattern raised suspicion of a non-communicating hydrocephalus pattern and possible proximal cerebrospinal fluid pathway obstruction. However, neither MRI nor CT could definitively localize the exact site or mechanism of obstruction because dedicated cerebrospinal fluid flow sequences and endoscopic evaluation were not available. Therefore, the findings should be interpreted as radiological suspicion rather than confirmation of secondary aqueductal obstruction. Congenital aqueductal stenosis appeared less likely based on the clinical evolution because ventricular dilatation developed after documented neonatal intraventricular hemorrhage; however, it could not be definitively excluded. The broad range of mechanisms underlying pediatric hydrocephalus further supports cautious interpretation of imaging findings when definitive anatomical confirmation is unavailable [10].

Cranial ultrasonography remains the preferred modality for monitoring ventricular size in preterm infants due to its safety and feasibility for serial assessment [4,5]. Serial cranial ultrasonography is particularly valuable because ventricular dimensions can be objectively assessed using established reference values [11]. In this patient, MRI had already demonstrated ventriculomegaly and sequelae of prior intraventricular hemorrhage. However, as the infant subsequently developed progressive macrocephaly, vomiting, poor feeding, a full anterior fontanelle, increased tone, and bilateral ankle clonus, CT was obtained as part of the neurosurgical evaluation of worsening hydrocephalus and possible need for intervention. CT provided rapid anatomical assessment of ventricular enlargement and cortical mantle thinning during a period of clinical deterioration. Its use in this case reflects real-world clinical decision-making rather than a recommendation for routine surveillance and differs from contemporary guideline-preferred approaches.

The selection of a ventricular tap as a temporizing intervention also reflects the complexity of real-world PHVD management. Current temporizing options include serial lumbar punctures, ventricular access devices, ventriculosubgaleal shunts, external ventricular drainage, and other center-dependent approaches [12,13]. Ventricular tapping is not generally considered a definitive treatment and is usually reserved for selected cases of acute clinical deterioration with signs of raised intracranial pressure [14]. In this patient, ventricular aspiration was performed because of progressive macrocephaly, vomiting, poor feeding, full fontanelle, increased tone, ankle clonus, and severe ventriculomegaly. Removal of approximately 60 mL of CSF resulted in transient clinical improvement, supporting its role as a temporary decompressive measure rather than definitive therapy.

Despite temporary improvement following ventricular tapping, progressive ventriculomegaly and continued enlargement of head circumference persisted, necessitating definitive cerebrospinal fluid diversion with ventriculoperitoneal shunt insertion. The patient subsequently developed VP shunt malfunction and central nervous system infection during follow-up. Cerebrospinal fluid cultures grew Staphylococcus lugdunensis, while CSF analysis demonstrated markedly elevated protein levels and low glucose concentrations, consistent with infection. The patient initially received intravenous ceftriaxone from 28 November to 5 December 2025 and was subsequently treated with culture-directed intravenous vancomycin (15 mg/kg/dose every 12 h) until 18 December 2025. Management also required surgical revision of the VP shunt because of partial obstruction of the ventricular catheter. This clinical course illustrates the complexity of long-term management in infants with shunt-dependent post-hemorrhagic ventricular dilatation, where both shunt-related complications and infection may necessitate repeated neurosurgical intervention. Previous studies, including the ELVIS trial, have emphasized that earlier intervention guided by ventricular measurements may reduce brain injury in selected infants with PHVD [15]. However, in clinical practice, management must be individualized according to neurological status, infection, shunt function, and local neurosurgical resources.

Emerging interventions such as drainage, irrigation and fibrinolytic therapy (DRIFT) and neuro-endoscopic lavage (NEL) aim to reduce the burden of intraventricular blood products and inflammatory debris in selected infants with PHVD [16,17,18,19]. These approaches may potentially reduce shunt dependency in carefully selected patients, but availability and experience vary considerably between centers. In the present case, management followed the locally available neurosurgical pathway, consisting of serial imaging surveillance, temporizing ventricular decompression, VP shunt placement, treatment of subsequent shunt-related infection, and shunt revision when malfunction occurred.

This case highlights several practical lessons. First, apparently low-grade IVH may still progress to severe PHVD, particularly when the initial hemorrhagic burden is underestimated or when progressive obstruction and inflammation evolve over time. Second, imaging patterns suggestive of non-communicating hydrocephalus should be interpreted cautiously unless confirmed by dedicated CSF flow studies or direct visualization. Third, temporizing interventions and definitive shunt placement must be individualized according to clinical deterioration, infection status, and available resources. Finally, long-term neurosurgical and neurodevelopmental follow-up is essential because shunt dependency and shunt-related complications remain important sources of morbidity in children with PHH [15,18]. In the present case, the subsequent need for ventriculoperitoneal shunt revision because of partial ventricular catheter obstruction further illustrates the complexity of long-term management in infants requiring permanent cerebrospinal fluid diversion. This emphasizes the importance of continued neurosurgical surveillance, as shunt malfunction remains a well-recognized source of morbidity in this population.

A limitation of this report is that the proposed mechanism of secondary cerebrospinal fluid pathway obstruction was inferred from the pattern of neuroimaging findings and the clinical evolution rather than being directly demonstrated. Although conventional brain MRI was performed during the clinical course, dedicated cerebrospinal fluid flow sequences were not obtained. In addition, endoscopic assessment and the original cranial ultrasonography images were unavailable for review. Consequently, the exact site and mechanism of obstruction could not be definitively established, and the imaging findings should be interpreted as suggestive of a non-communicating hydrocephalus pattern rather than definitive evidence of obstruction. Furthermore, opening and closing intracranial pressure measurements during ventricular tapping were not documented. Finally, long-term neurodevelopmental outcome data beyond the available follow-up period were unavailable, limiting assessment of the eventual neurological impact of the ventricular dilatation and subsequent interventions.

## 4. Patient Perspective

The infant’s parents described the prolonged hospital course and the need for repeated neurosurgical interventions as a significant source of anxiety. They expressed appreciation for the regular communication provided by the multidisciplinary team and understood the need for continued long-term follow-up because of the possibility of future shunt-related complications and neurodevelopmental concerns.

## 5. Conclusions

This case illustrates the atypical progression of an initially documented Grade II intraventricular hemorrhage to severe post-hemorrhagic ventricular dilatation requiring temporizing cerebrospinal fluid diversion and definitive ventriculoperitoneal shunting. Serial neuroimaging demonstrated progressive ventriculomegaly, while computed tomography showed an imaging pattern suggestive—but not diagnostic—of a non-communicating hydrocephalus pattern, emphasizing the importance of cautious radiological interpretation.

The clinical course highlights the challenges of managing post-hemorrhagic ventricular dilatation in extremely premature infants, particularly with respect to ongoing neuroimaging surveillance, selection of temporizing interventions, and timing of definitive cerebrospinal fluid diversion in the setting of intercurrent infection. This case demonstrates that apparently low-grade intraventricular hemorrhage may still progress to severe shunt-dependent hydrocephalus and underscores the need for individualized multidisciplinary management and careful longitudinal follow-up.

## Figures and Tables

**Table 1:** Clinical timeline of disease progression and management.

Age/Timepoint	Clinical Event
Birth (26 weeks’ gestation)	Extreme prematurity (birth weight 1.22 kg); NICU admission for respiratory distress requiring surfactant therapy and ventilatory support
14 April 2025 (Day 3 of life)	Cranial ultrasonography demonstrated **Grade II intraventricular hemorrhage (IVH)** with associated intracerebral hematoma
9 May 2025	Follow-up cranial ultrasonography demonstrated **progressive ventricular dilatation** with enlargement of the lateral, third, and fourth ventricles
9 June 2025	Serial ultrasonography showed **persistent ventriculomegaly** without significant interval improvement
29 September 2025 (approximately 50 weeks PMA)	Head circumference increased from **26 cm on Day 3 of life** to approximately **40 cm**, with progressive macrocephaly and severe ventricular dilatation on serial imaging
29 September 2025 (approximately 50 weeks PMA)	CT brain demonstrated marked enlargement of the lateral and third ventricles with severe cortical mantle thinning and relative preservation of the fourth ventricle
29 September 2025 (approximately 50 weeks PMA)	Irritability, poor feeding, intermittent vomiting, full anterior fontanelle, increased tone, and bilateral ankle clonus suggesting raised intracranial pressure
30 September 2025 (approximately 50 weeks PMA)	Ventricular tap performed as a **temporizing cerebrospinal fluid diversion** procedure with approximately **60 mL CSF removed**, resulting in transient clinical improvement
6 October 2025	Ventriculoperitoneal (VP) shunt inserted as definitive cerebrospinal fluid diversion because of persistent progressive ventriculomegaly, increasing head circumference, and clinical signs of raised intracranial pressure
26 November 2025	Re-admitted with progressive irritability, increasing head circumference, and concern for VP shunt malfunction/infection Cerebrospinal fluid analysis demonstrated glucose **1.2 mmol/L**, protein **2175 mg/L**, and positive culture for *Staphylococcus lugdunensis*, consistent with central nervous system infection. VP shunt revision performed; operative findings demonstrated partial obstruction of the ventricular catheter requiring revision of the cranial and abdominal shunt components.
28 November 2025 **16 December 2025**	Discharged clinically stable with functioning revised VP shunt. Follow-up arranged with neurosurgery and developmental pediatrics.

## Data Availability

The data presented in this study are available on request from the corresponding author. The data are not publicly available due to patient privacy and ethical restrictions.
